# Short-term tranexamic acid treatment reduces in-hospital mortality in aneurysmal sub-arachnoid hemorrhage: A multicenter comparison study

**DOI:** 10.1371/journal.pone.0211868

**Published:** 2019-02-07

**Authors:** R. Post, M. R. Germans, H. D. Boogaarts, B. Ferreira Dias Xavier, R. Van den Berg, B. A. Coert, W. P. Vandertop, D. Verbaan

**Affiliations:** 1 Neurosurgical Center Amsterdam, Amsterdam UMC, Univ(ersity) of Amsterdam, Amsterdam, the Netherlands; 2 Department of Neurosurgery, Clinical Neuroscience Center, University Hospital Zürich, Zürich, Switzerland; 3 Department of Neurosurgery, Radboud University Medical Center, Nijmegen, the Netherlands; 4 Faculty of Medicine, University do Rio Grande do Norte, Lagoa Nova, Natal, Brazil; 5 Department of Radiology, Amsterdam UMC, Univ(ersity) of Amsterdam, Amsterdam, the Netherlands; Universitatsklinikum Freiburg, GERMANY

## Abstract

**Background:**

Recurrent bleeding is one of the major causes of morbidity and mortality in patients with aneurysmal subarachnoid hemorrhage (aSAH). Antifibrinolytic therapy is known to reduce recurrent bleeding, however, its beneficial effect on outcome remains unclear. The effect of treatment with tranexamic acid (TXA) until aneurysm treatment on clinical outcome is evaluated.

**Methods:**

Patients with an aSAH from two high-volume tertiary referral treatment centers in the Netherlands, Academic Medical Center (AMC) and Radboud University Medical Center (RUMC), between January 2012 and December 2015 were included. Patients were classified into one of two groups; standard treatment or TXA treatment. Demographic and clinical characteristics, in-hospital complications and clinical outcome were compared between the two groups. Multivariate logistic regression was used to adjust for the influence of treatment center and baseline differences.

**Results:**

Standard treatment was given in 509 patients, and 119 patients received additional TXA therapy before aneurysm occlusion. Patients treated with TXA did not experience less recurrent bleeding adjusted or unadjusted for treatment center (adjusted odds ratio [aOR] 0.80, 95% confidence interval [95% CI]: 0.37–1.73). In-hospital mortality, was significantly lower in the TXA group than the standard care group (adjusted OR [aOR] 0.42, 95% CI: 0.20–0.85). Poor outcome (mRS 4–6) assessed after six months was not different between treatment groups (aOR 1.05, 95% CI: 0.64–1.74).

**Conclusions:**

Pooled data from two high-volume treatment centers did not show improved clinical outcome after additional TXA treatment in aSAH patients. However, TXA treatment was associated with a decrease in mortality.

## Introduction

In the last decades, there is an increasing trend towards ultra-early (< 24 hours) aneurysm occlusion to protect from recurrent bleeding after aneurysmal subarachnoid hemorrhage (aSAH), as most recurrent bleedings occurs within a few hours after the initial bleeding[1], [2], [3], [4], [5], [6]. In clinical practice, however, early treatment can be logistically challenging, and therefore, is frequently delayed [7]. Based on Class IIa, level B evidence, the current AHA/ASA guideline states that “short-term (<72 hours) therapy with tranexamic acid or aminocaproic acid is reasonable to reduce the risk of early aneurysm rebleeding”, and therefore, despite a recent inconclusive Cochrane Database Systematic Review, some centers have incorporated tranexamic acid (TXA) into their aSAH treatment protocols[8], [9], [10].

Inquiries among high-volume aSAH treatment centers in the Netherlands for participation in a currently ongoing multicenter, randomized controlled trial[11] (RCT) revealed that one treatment center included TXA administration prior to aneurysm occlusion in their protocol for patients admitted to the Intensive Care Unit. To investigate whether TXA treatment improves clinical outcome in a larger patient population, two prospective databases from two university medical centers in the Netherlands were pooled and analyzed.

## Methods

### Patient population

Adult patients with aSAH from a prospective database, who were admitted between January 2012 and December 2015 to the Academic Medical Center (Amsterdam, the Netherlands; AMC) and Radboud University Medical Center (Nijmegen, the Netherlands; RUMC), both high-volume tertiary care centers, were included in this study if 1) subarachnoid hemorrhage (SAH) was confirmed by a plain CT-scan on admission, or by presence of xanthochromia in the cerebral spinal fluid, 2) a causative aneurysm was documented by CT-angiography (CTA) and/or digital subtraction angiography (DSA), 3) onset of symptoms to admission was within 24 hours. Patients with SAH in whom an intracranial aneurysm could not be visualized, and patients participating in an ongoing RCT were excluded from this analysis. The AMC patients did not receive TXA, whereas the RUMC treatment protocol included TXA treatment before aneurysm occlusion. Patients were classified into one of two groups (standard vs. TXA), based on treatment with TXA.

### Data collection

The results were reported according to the STROBE guidelines[12]. At baseline, the following items were collected from our prospective database: demographic characteristics, World Federation of Neurosurgical Societies (WFNS) grade[13] on admission at the treatment center, Fisher grade[14]. Furthermore, location of aneurysm, treatment modality (i.e. coiling or clipping), time intervals (ictus-treatment, ictus-hospital admission, hospital admission-aneurysm treatment), recurrent bleeding, per-procedural complications, systemic thromboembolic complications, delayed cerebral ischemia (DCI), hydrocephalus, implantation of permanent shunt for cerebrospinal fluid diversion, in-hospital mortality, length of hospital stay (LOS), discharge location and clinical outcome assessed after six months by the modified Rankin Scale (mRS), using a standardized telephone interview by experienced specialized nurses, or on doctors’ experience and patient-reported functioning. Additionally, we performed a retrospectively chart review to collected daily and total dosage of administered TXA. The study protocol was approved by the local IRB’s and granted a waiver of consent.

Recurrent bleeding was defined as a second (or third etc.) bleeding from the causative aneurysm after the initial bleeding. This was determined by an increase of blood on a plain CT-scan of the head. Delayed cerebral ischemia was defined according to Vergouwen et. al.[15]. Hydrocephalus was defined either by enlarged ventricles on imaging, assessed by an experienced neuroradiologist, or by increased intracranial pressure diagnosed by lumbar puncture or ventricular catheter placement.

### Treatment and clinical management

All patients were treated according to their hospital’s standardized protocols, which were based mainly on the International Guidelines of 2009, including calcium antagonists (Nimodipine), and hypertensive therapy and normovolemia when DCI was suspected; no rescue treatment was initiated when hypertensive therapy failed. Blood pressure was lowered until aneurysm occlusion using administration of short-acting, titratable, continuous intravenous (IV) labetalol, if the mean arterial pressure was above 110mmHg and 135mmHg in the RUMC and AMC, respectively. Patients who were treated with TXA received a loading dose of one gram intravenously on admission to the emergency room or neurosurgical intensive care unit of the RUMC, immediately followed by 1 gram as a continuous infusion over 8 hour intervals, until occlusion of the causative aneurysm, or until a maximum of 36 hours. TXA treatment was withheld in case of severe renal impairment (serum creatinin > 150 mmol/L. No TXA was administered when patients went for aneurysm treatment immediately after presentation to the emergency room. Ruptured aneurysms were treated as early as feasible (preferably within 24 hours after onset). An external ventricular or lumbar catheter was placed for CSF drainage in case of hydrocephalus, or if raised intracranial pressure was suspected.

All patients were treated during admission with prophylactic nadroparin and compression stockings until discharge.

### Statistical analysis

The WFNS grades and mRS scores were dichotomized into good (WFNS grade 1–3) or poor grade (WFNS grade 4–5), and into favorable (mRS 0–3) or poor (mRS 4–6) outcome. All other endovascular treatments, i.e. web device, additional stenting or flow-diverter were classified into coiling. Fisher grades were dichotomized into grade 0–3 and grade 4. Both prospective databases (AMC / RUMC) were pooled and patients were allocated into one of two groups, based on TXA treatment. Normally distributed variables, tested with the Shapiro Wilk test (>0.9 is normally distributed), were expressed as means with standard deviations (SD) and tested with the Student’s T test, and unequally distributed variables as medians with interquartile ranges (IQR 25–75%) and tested with the Mann–Whitney U test. The Chi-square or Fisher’s exact test was used to assess differences in proportions wherever appropriate. Multivariate logistic regression was used to take the combination of treatment center and TXA administration into account (three groups: AMC control, RUMC control, and RUMC TXA) and to adjust for baseline differences. P-values < 0.05 were considered significant. Statistical analyses were performed using the SPSS Statistics Software (IBM Corporation, New York, United States, version 21).

## Results

### Baseline demographics

**Fig 1:**
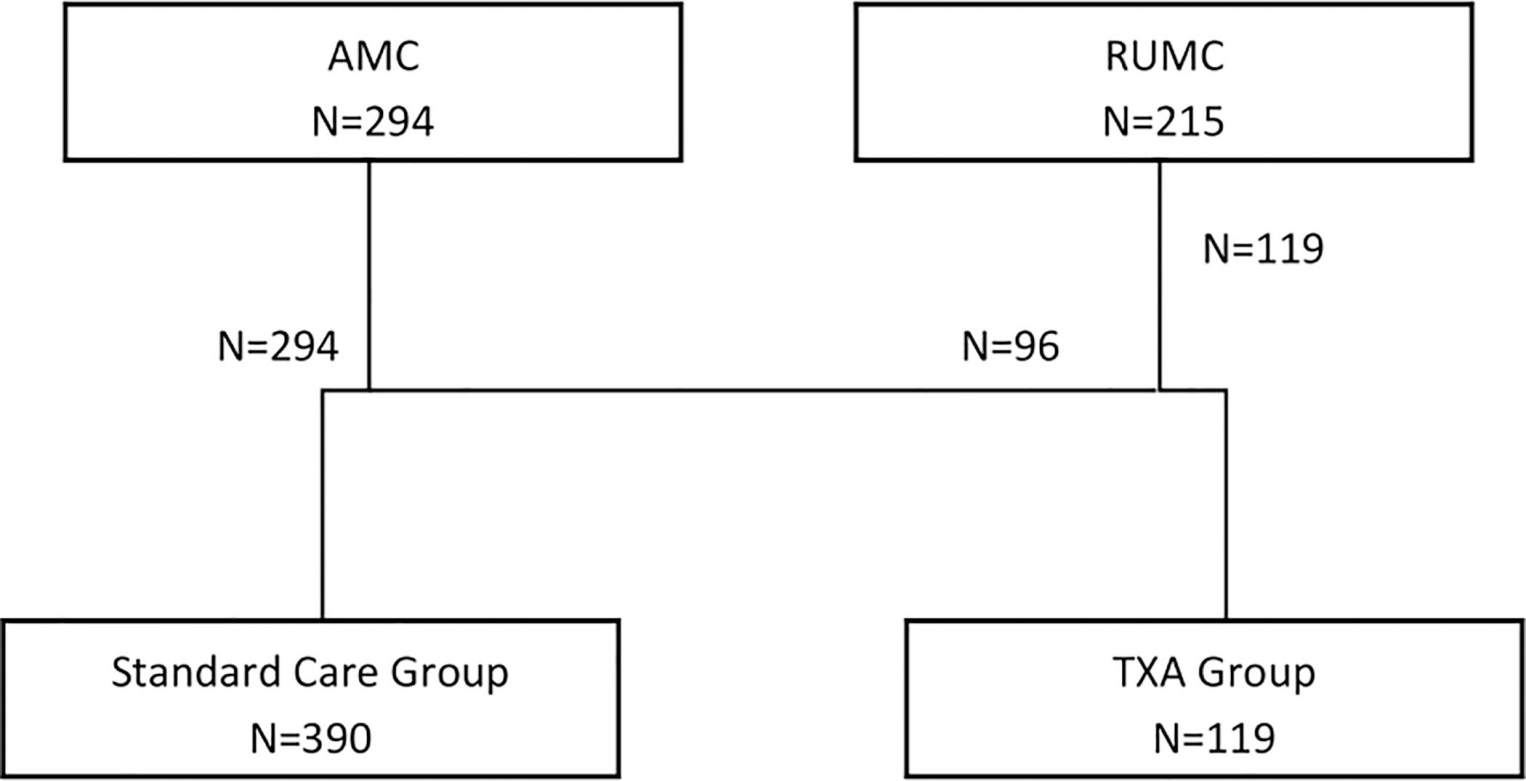
Flowchart allocation per treatment group.

Between January 2012 and December 2015, a total of 509 patients with an aSAH that met the inclusions criteria, were admitted (Fig 1). In 390 (77%) patients no TXA was given (standard treatment group, RUMC, n = 96; AMC, n = 294), whereas 119 (23%) patients received additional TXA treatment (TXA group, all RUMC).

Overall baseline data (Table 1) did not differ significantly between both groups.

**Table 1:** Baseline demographics of 509 patients with aneurysmal subarachnoid hemorrhage.

Characteristic	TXA Group (%) N = 119	Standard Group (%) N = 390	Total (%) N = 509	OR (95% CI)
**Age** in years, *mean (SD)*	58 (12)	57 (13)	57 (13)	1.01 (0.99–1.03)
**Female**	84 (71)	268 (69)	352 (70)	1.09 (0.70–1.71)
WFNS Grade^*^				1.07 (0.71–1.63)
I-III	69 (58)	231 (60)	300 (59)	
IV-V	50 (42)	156 (40)	206 (41)	
Fisher Grade Scale^*^				1.01 (0.71–1.45)
0–3	32 (27)	104 (27)	136 (27)	
4	86 (73)	285 (73)	371 (73)	
**Causative aneurysm** location^*^				0.98 (0.71–1.36)
Anterior circulation	75 (64)	245 (63)	320 (63)	
Posterior circulation	43 (36)	144 (37)	187 (37)	
**Aneurysm treatment**				1.16 (0.86–1.55)
None	12 (10)	61 (16)	73 (14)	
Coiling	87 (73)	278 (71)	365 (72)	
Clipping	20 (17)	51 (13)	71 (14)	
**Time intervals (hours)**				
aSAH to admission	2 (1–6)	3 (1–5)	3 (1–5)	1.02 (0.98–1.06)
aSAH to treatment^%^	13 (8–21)	9 (3–16)	9 (3–16)	1.00 (1.00–1.00)
Admission to treatment^^^	13 (8–21)	12 (6–22)	13 (6–22)	1.00 (1.00–1.00)
aSAH to start TXA	5 (3–11)	-	5 (3–11)	-

SD = Standard deviation

*based on *n =* 507, ^%^based on *n =* 410, ^^^based on *n =* 423, *N* (%) unless otherwise stated.

The overall median time interval between onset of SAH and admission to treatment center was three hours (IRQ 1–5), and was not significantly different between treatment groups.

### Complications

Overall recurrent bleeding was 14% and occurred as often in the TXA group as in the standard care group (13% and 14%, respectively). Procedural thrombus formation occurred slightly more in the TXA group (12% in the TXA group and 9% in the standard group), not significant different.

No significant differences were seen in other per-procedural complications (rupture, treatment ischemia). Use of TXA did not lead to a significantly increased rate of DCI, hydrocephalus, or the need for a permanent shunt (Table 2). No systemic thromboembolic complications occurred in the TXA group whereas three systemic thromboembolic complications occurred in the standard group (one myocardial infarct, one atrial thrombosis and one pulmonary embolism). There were also no significant differences between groups if complications were compared between three groups (RUMC TXA, RUMC controls, and AMC controls).

**Table 2:** Complications per treatment group and adjusted per center with or without TXA treatment.

	TXA Group N = 119	Standard Group N = 390	Total N = 509	OR (95% CI)	aOR (95% CI)
**Recurrent bleeding** (N = 500)	15 (13)	53 (14)	68 (14)	0.97 (0.52–1.79)	0.80 (0.37–1.73)
**Procedural rupture** (N = 423)	3 (3)	11 (3)	14 (3)	0.83 (0.23–3.04)	0.45 (0.11–1.95)
**Delayed cerebral ischemia** (N = 503)	21 (18)	95 (25)	116 (23)	0.68 (0.40–1.15)	0.99 (0.49–2.00)
**Treatment ischemia** (N = 426)	4 (4)	15 (5)	19 (5)	0.82 (0.27–2.52)	1.58 (0.28–8.85)
**Procedural thrombus** (N = 425)	12 (12)	28 (9)	40 (9)	1.37 (0.67–2.79)	1.63 (0.58–4.55)
**Hydrocephalus** (N = 476)	63 (62)	223 (560)	286 (60)	1.13 (0.72–1.78)	1.33 (0.73–2.41)

*N* (%) unless otherwise stated.

### Clinical outcome

In-hospital mortality was significantly lower in the TXA group (TXA group: 16%, standard group: 25%; OR 0.57; 95% CI: 0.33–0.98). Poor outcome (mRS 4–6) at six months was not significantly different after adjusting and before adjusting for treatment centers and TXA treatment (TXA group: 34%, standard group: 40%; OR 0.76; 95% CI: 0.48–1.18) (Table 3). There were also no significant differences between groups if poor outcome was compared between three groups (RUMC TXA, RUMC controls, and AMC controls).

**Table 3:** Patient outcome by treatmentgroup and adjusted per center with or without TXA treatment.

	TXA Group (%) N = 119	Standard Group (%) N = 390	Total (%) N = 509	OR (95% CI)	aOR (95% CI)
**In hospital mortality** (N = 509)	19 (16)	97 (25)	116 (23)	**0.57 (0.33–0.98)**	**0.42 (0.22–0.80)**
**Poor outcome** (N = 481)	37 (34)	149 (40)	186 (39)	0.76 (0.48–1.18)	0.81 (0.45–1.44)
**mRS 4 and 5** (N = 481)	14 (16)	44 (17)	58 (16)	0.97 (0.50–1.87)	2.59 (0.81–8.31)
**LOS** (days)^#^ (N = 386)	16 (11–29)	17 (11–26)	16 (11–27)	1.00 (0.99–1.01)	1.02 (0.99–1.04)
**Discharge location**^**#**^ (N = 389)					
Home	36 (37)	119 (41)	155 (40)	0.84 (0.52–1.35)	0.74 (0.39–1.40)
Other hospital	34 (35)	120 (41)	154 (40)	0.76 (0.47–1.22)	0.87 (0.46–1.67)
Rehabilitation	12 (12)	35 (12)	47 (12)	1.02 (0.51–2.06)	0.70 (0.29–1.69)
Nursing home	15 (15)	13 (5)	28 (7)	3.86 (1.77–8.45)	N/A*
Other	1 (1)	4 (1)	5 (1)	0.74 (0.08–6.70)	N/A*
**Permanent Shunt**^**#**^ (N = 467)	18 (19)	46 (12)	64 (14)	1.68 (0.93–3.07)	1.66 (0.72–3.83)

*N* (%) unless otherwise stated.

*adjustments could not be made due to the low number of patients.

# numbers based on survivors

## Discussion

This study in patients with aneurysmal SAH compared treatment with TXA until aneurysm repair to standard treatment, based on two pooled, prospective databases from high-volume treatment centers in the Netherlands, with comparable treatment protocols. After adjusting for treatment center and treatment, a significant decrease in in-hospital mortality was seen in patients who were treated with TXA and poor outcome after six months was lower in patients treated with TXA, compared to the standard treatment group, although without a statistical difference.

Almost all recurrent bleedings occur within 24 h, with a median time interval between initial hemorrhage and recurrent bleeding of 180 min[1]. We recently showed that mortality was significantly higher in patients with a recurrent bleeding, with less patients achieving a favorable outcome[1]. Thus, early occlusion of a ruptured aneurysm seems warranted. However, because a large number of recurrent bleedings occurs in the first few hours, aneurysm occlusion needs to be ultra-early, which remains challenging, especially when patients need to be transferred to specialized treatment centers[16]. To overcome the problem of ultra-early recurrent bleedings, antifibrinolytic agents, such as TXA or epsilon-aminocaproic acid (EACA), have been extensively investigated in the past. Although the recurrent bleeding risk is reduced by TXA treatment, a concomitant increase in delayed ischemic deficits and thrombotic complications leads to no significant net improvement in clinical outcome[10], [17]). The study by Hillman[9] with short-term (up to 72 hours) treatment with antifibrinolytic therapy, however, showed promising results with less recurrent bleedings and no significant increase in ischemic neurological deficits. To date, the controversy remains regarding a beneficial effect on clinical outcome of antifibrinolytic agents.

Our study differs from previously reported studies, because it combines two high volume treatment centers with comparable SAH treatment protocols, but different approaches to the use of TXA. The strength of our study is that we increased power by pooling data of large treatment centers with comparable treatment protocols with different use of TXA, making our results more generally interpretable. Furthermore, due to the collection of intervals between ictus-admission and time to treatment the two treatment protocols could be more exactly compared, especially in respect to “preventable” early recurrent bleeding with TXA.

Surprisingly, TXA treatment did not significantly reduce recurrent bleedings in our study. This could be explained by the fact that TXA treatment was only started after arrival in both treatment centers, whereas most recurrent bleeding events occurred during transport or when patients were still in the referring hospital as almost half of all recurrent bleedings are known to occur within the first three hours after diagnosis[1]. It could also be caused by lack of power because recurrent bleeding is relatively. We found that TXA treatment was associated with a significant decrease in mortality. Furthermore TXA treatment showed a trend towards an increase in favorable outcome, which is comparable to previous studies that administered antifibrinolytic agents early and for a maximum of 72 hours[18]. This demonstrates that besides short-term treatment with antifibrinolytics, as previously promoted in the literature[19], [18], an ultra-early TXA treatment protocol could potentially lead to better outcomes.

To address this question therefore, a multicenter randomized controlled trial[11] (RCT) was developed and is currently enrolling patients.

Noteworthy, with this short-term TXA treatment, there was no increase in the occurrence of ischemic or thromboembolic complications, nor was there an increase in delayed cerebral ischemia, in accordance to the recent literature [20]. In our study we found a low rate of systemic thromboembolic complications compared to Foreman et.al.[17] and Starke et. al.[18]. All our patients were treated with prophylactic anticoagulation for DVT with additional compression stockings with no routine screening for asymptomatic DVT.

The main limitations of our study are, first of all, that TXA administration times were collected retrospectively, although all other patient data was collected prospectively. Secondly, although the center that had additional treatment with TXA in its protocol, only two third of the patients were actually treated with TXA, leading to a ratio (TXA vs standard group) of one to three, and although no significant baseline differences were found, this might have introduced a selection bias due to physician selection of initiation of additional TXA treatment. Thirdly, in our cohort, recurrent bleeding percentages were comparable in both groups. One explanation for the lack of reduction in recurrent bleeding is, that TXA treatment was started only in the treatment center and not as early as possible in the referring hospital, thus exposing patients to ultra-early recurrent bleeding during stay in referring hospitals or during transfers. Although recurrent bleeding were similar we found a significantly decrease in mortality in the TXA group due to less severe recurrent bleeding. Unfortunately no volume data on recurrent bleeding was collected thus this remains a hypothetical explanation but should be addressed in further studies. Lastly, modified Rankin scale scores were not scored in the same standardized fashion in both centers. One of the treatment centers performed the standardized and validated telephone interview by trained and experienced research nurses, whereas the other center based the mRS score at six months on doctors’ experience and patient-reported functioning, as noted in the electronic patient records. Therefore, interpretation differences could potentially have biased our results. However, by dichotomizing the mRS into good (mRS 0–3) and poor (mRS 4–6), this interpretation bias should be minimal.

## Conclusion

We found that TXA treatment was associated with a decrease in mortality and showed a trend towards a better clinical outcome after six months in the group treated with short-term TXA. No increase in thromboembolic complications was seen in TXA treatment. Evaluation of ultra-early (at first diagnosis) and short-term (<24 h) TXA administration in a randomized larger population is needed to assess the beneficial effect on outcome in aSAH patients.

## Abbreviations

aSAH: aneurysmal subarachnoid hemorrhage
aOR: adjusted OR
DCI: delayed cerebral ischemia
CSF: cerebral spinal fluid
CT: computed tomography
95% CI: 95% confidence interval
DSA: digital subtraction angiography
SD: standard deviations
OR: odds ratio
TXA: tranexamic acid
WFNS: World Federation of Neurological Surgeons
